# Bestandsaufnahme der verfügbaren und aktuell eingesetzten Typisierungsmethoden einschließlich genombasierter Verfahren von Zoonoseerregern am Beispiel von *Salmonella enterica*

**DOI:** 10.1007/s00103-022-03622-y

**Published:** 2022-12-22

**Authors:** Michael Pietsch, Sandra Simon, Anne Richter, Burkhard Malorny, Laura Uelze, Sabrina Hepner, Alexandra Dangel, Andreas Sing, Ingrid Huber, Ulrich Busch, Jörg Linde, Ulrich Methner, Natalie Becker, Guido Werner, Alexander Mellmann, Angelika Fruth, Antje Flieger

**Affiliations:** 1 Fachgebiet 11: Bakterielle darmpathogene Erreger und Legionellen und Nationales Referenzzentrum (NRZ) für Salmonellen und andere bakterielle Enteritiserreger, Robert Koch-Institut (RKI), Burgstr. 37, 38855 Wernigerode, Deutschland; 2 Abteilung Biologische Sicherheit, Bundesinstitut für Risikobewertung (BfR), Berlin, Deutschland; 3 Bayrisches Landesamt für Gesundheit und Lebensmittelsicherheit, Oberschleißheim, Deutschland; 4 Institut für bakterielle Infektionen und Zoonosen, Friedrich-Loeffler-Institut (FLI), Jena, Deutschland; 5 Referat für Krisenmanagement und Lebensmittelbetrug, Bundesamt für Verbraucherschutz und Lebensmittelsicherheit (BVL), Berlin, Deutschland; 6 Fachgebiet Nosokomiale Infektionserreger und Antibiotikaresistenzen, Robert Koch-Institut (RKI), Wernigerode, Deutschland; 7 Institut für Hygiene und Nationales Konsiliarlaboratorium für Hämolytisch-Urämisches Syndrom (HUS), Universitätsklinikum Münster, Münster, Deutschland

**Keywords:** Molekulare Epidemiologie, WGS, Molekulare Surveillance, Salmonella enterica, Public Health, Molecular epidemiology, WGS, Molecular surveillance, Salmonella enterica, Public health

## Abstract

**Hintergrund:**

In den vergangenen Jahren hat sich die Gesamtgenomsequenzierung („whole genome sequencing“; WGS) in Kombination mit bioinformatischen Analysen zum Stand der Technik bei der Bewertung des Pathogenitäts- und Resistenzpotenzials sowie der Verwandtschaftsgrade zwischen Bakterien entwickelt. Die WGS-Analyse stellt somit ein zentrales Instrument bei der Typisierung von Erregern und der Untersuchung von Krankheits- und Ausbruchsclustern im Rahmen der molekularen Epidemiologie dar. Ziel der Studie war die Generierung eines Überblicks der in Deutschland auf Landes- und Bundesebene verfügbaren Erregertypisiermethoden von Salmonellen und Shiga-Toxin-bildenden bzw. enterohämorrhagischen *Escherichia coli* (STEC/EHEC) und den angewandten geno- und phänotypischen Methoden sowie über die Verfügbarkeit der genombasierten Typisierung und entsprechenden Analyseverfahren.

**Methoden:**

Im Zeitraum vom Februar bis Juni 2020 wurde eine elektronische Umfrage bei Laboratorien durchgeführt, die für den öffentlichen Gesundheitsschutz und gesundheitlichen Verbraucherschutz tätig sind.

**Ergebnisse und Fazit:**

Die Ergebnisse der Umfrage zeigten, dass viele der teilnehmenden Laboratorien über eine große Auswahl an phänotypischen und molekularbiologischen Methoden verfügen. Molekularbiologische Typisierungen werden am häufigsten für die Speziesidentifizierung von Salmonellen herangezogen. WGS-Verfahren sind vielfach schon bei Einrichtungen auf Bundes- und Landesebene etabliert oder befinden sich im Aufbau. Die Illumina-Sequenzierung ist dabei die am weitesten verbreitete Technologie. Die Umfrage bestätigt die Bedeutung von molekularbiologischen und genombasierten Typisierungstechnologien für die Laboratorien bei der Diagnostik von bakteriellen zoonotischen Erregern.

## Abstract (en)

**Background:**

In recent years, whole genome sequencing (WGS) in combination with bioinformatic analyses has become state of the art in evaluating the pathogenicity/resistance potential and relatedness of bacteria. WGS analysis thus represents a central tool in the investigation of the resistance and virulence potential of pathogens, as well as their dissemination via outbreak clusters and transmission chains within the framework of molecular epidemiology. In order to gain an overview of the available genotypic and phenotypic methods used for pathogen typing of *Salmonella* and Shiga toxin-producing and enterohemorrhagic *Escherichia coli* (STEC/EHEC) in Germany at state and federal level, along with the availability of WGS-based typing and corresponding analytical methods, a survey of laboratories was conducted.

**Methods:**

An electronic survey of laboratories working for public health protection and consumer health protection was conducted from February to June 2020.

**Results and conclusion:**

The results of the survey showed that many of the participating laboratories provide a wide range of phenotypic and molecular methods. Molecular typing is most commonly used for species identification of *Salmonella*. In many cases, WGS-based methods have already been established at federal and state institutions or are in the process of being established. The Illumina sequencing technology is the most widely used technology. The survey confirms the importance of molecular biology and whole genome typing technologies for laboratories in the diagnosis of bacterial zoonotic pathogens.

## Einleitung und Hintergrund

In den vergangenen Jahren hat sich die Gesamtgenomsequenzierung („whole genome sequencing“; WGS) in Kombination mit bioinformatischen Analysen zum Stand der Technik bei der Bewertung des Pathogenitäts‑/Resistenzpotenzials von Bakterien entwickelt. Die WGS-Analyse stellt somit ein zentrales Instrument bei der Untersuchung von Erregern und ihren Krankheitsclustern im Rahmen der molekularen Epidemiologie dar [1, 2].

Gerade bei zoonotischen Erregern, die über Lebensmittel auf den Menschen übertragen werden, spielt die sektorübergreifende Analyse der Erreger eine besonders große Rolle, d. h. die gemeinsame Analyse und Typisierung der Erreger durch Einrichtungen des Öffentlichen Gesundheitsdiensts (ÖGD) und der Lebensmittelüberwachung (LÜ).

Neben dem Nachweis des Erregers beim Erkrankten oder dem Lebensmittel spielt die Feintypisierung zur Unterscheidung von sehr ähnlichen Erregertypen eine große Rolle bei der Erkennung von Krankheitsclustern und deren Lebensmittelquellen. Im Gegensatz zu bis in die letzten Jahre standardmäßig genutzten Feintypisierungsmethoden, wie Multiple Locus-Variable-Number-Tandem-Repeat-Analyse (MLVA) oder Makrorestriktionsanalyse mit anschließender Pulsfeld-Gel-Elektrophorese (PFGE), bietet die WGS neben dem hohen Grad an Reproduzierbarkeit den Vorteil eines viel höheren Auflösungsvermögens [3]. Damit wird eine präzisere Typisierung und, in Kombination mit weiteren (epidemiologischen) Daten wie Probenahmeort, -zeit und Isolationsmatrix, die Identifizierung von Infektionsquellen und Übertragungswegen von Erregern möglich sowie darüber hinaus eine Bewertung des Pathogenitäts- und Resistenzpotenzials [4, 5].

Die WGS und die anschließenden bioinformatischen Analysen sind in den vergangenen Jahren immer kostengünstiger und durch viele verschiedene verfügbare nutzerfreundliche Softwareprogramme einfacher zugänglich geworden. Trotzdem stellt die Implementierung dieser Technologie eine Herausforderung dar, vor allem durch den initial hohen Aufwand (hohe Startkosten, Etablierungs- und Schulungsaufwand), die Notwendigkeit des Aufbaus bioinformatischer Expertise zur Auswertung der Daten und die rechtlichen Unsicherheiten (Umgang mit WGS-Daten: Weitergabe, Aufbewahrungsdauer).

Während Umfragen auf europäischer Ebene zeigten, dass immer mehr Laboratorien in den einzelnen Ländern Zugang zu WGS und einer Auswertung erhobener Daten haben [6], ist es von großer Bedeutung, den Stand der Laboratorien auf nationaler Ebene zu erfassen, deren Entwicklungsbedarf abzuleiten und bei Bedarf zur Anwendung einer WGS-basierten Erregertypisierung zu ertüchtigen.

Im Jahr 2018 wurden zum Ausbau der genombasierten Surveillance vom Bundesministerium für Gesundheit (BMG) Projekte im Themenfeld „Integrierte genombasierte Surveillance von zoonotischen Erregern und Erregern mit speziellen Antibiotikaresistenzen“ ausgeschrieben. Aus diesem Aufruf haben sich 3 Forschungskonsortien konstituiert, welche Modelle/Konzepte für eine integrierte genombasierte Surveillance erarbeiten, die auf hochauflösenden Genomsequenzierverfahren basieren (siehe Infobox 1). Bei den 3 Konsortien handelt es sich um die Projekte *Integrierte genombasierte Surveillance von Salmonellen* (GenoSalmSurv), *Integrierte Genomische Surveillance von Zoonoseerregern* (IGS-Zoo) und *Genombasierte Surveillance übertragbarer Colistin- und Carbapenemresistenzen Gram-negativer Infektionserreger* (GÜCCI). Am Beispiel der Erreger *Salmonella enterica* und Shiga-Toxin-bildenden bzw. enterohämorrhagischen *Escherichia coli* (STEC/EHEC) sollen Modelle für eine integrierte genomische Surveillance von Lebensmittelpathogenen entwickelt und verfügbar gemacht werden.

Die nachfolgende Umfrage wurde gestaltet, um in einer frühen Projektphase in den Jahren 2019/2020 bei Laboratorien auf nationaler Ebene einen Überblick zu gewinnen über die verfügbaren Erregertypisiermethoden von Salmonellen und STEC/EHEC, die angewandten geno- und phänotypischen Methoden sowie die WGS-basierte Typisierung und entsprechende Analyseverfahren von bakteriellen Erregern. Ziel ist es, die in der Umfrage erlangten Ergebnisse in der Erstellung von Standardprotokollen, dem Aufbau einer Ressort-übergreifenden Surveillance (Mensch, Tier, Lebensmittel) von zoonotischen Infektionserregern mittels WGS-Analyse und in Schulungskonzepte für diese Thematik miteinzubeziehen.

## Methoden

### Ausrichtung der Umfrage und technische Umsetzung

Für die Erhebung wurde ein elektronischer Fragebogen mit Hilfe der Software Acuity4Survey (6.0.0.51, Voxco, Montreal, Canada) entwickelt. Im Rahmen des Fokus der Forschungsverbünde GenoSalmSurv, IGS-Zoo und GÜCCI wurde der Schwerpunkt auf die Erhebung von verfügbaren und tatsächlich angewandten Typisierungsmethoden sowie dem Zugang zu und der Anwendung von Genomsequenzierungstechnologien gelegt.

Der Aufbau der Umfrage und die Konzeption der Fragen wurden in mehreren Feedback-Runden mit den Konsortien etabliert. Der Fragebogen enthält 5 Hauptthemen: „Allgemeine Labor‑/Institutsinformationen“ (I), „phäno- und genotypische Methoden“ (differenziert in *Salmonella* [II_a_] und STEC/EHEC [II_b_]), „Verfügbarkeit und Durchführung von Genomsequenzierung“ (III), „Bioinformatische Auswertung von WGS-Daten“ (IV) und „Schulungsbedarf“ (V). Basierend auf der Erregerfilterfrage wurden der Umfang und die Art der Fragen des Fragebogens für die Teilnehmenden differenziert. Die Antworten wurden hauptsächlich als Einzel‑/Mehrfachoptionen aus einem Satz vordefinierter Antworten gesammelt, ermöglicht wurde auch die optionale Eingabe von Freitext. Diese qualitativ offenen Antwortmöglichkeiten wurden aufgenommen, um Kontext zu den Antworten zu gewinnen.

Hauptsächliche Zielgruppe der Befragung waren Laboratorien im Bereich der staatlichen Untersuchungsämter im Veterinärwesen, der LÜ und des ÖGD der Länder sowie vergleichbare Einrichtungen des Bundes. Weiterhin wurden auch ausgewählte universitäre Einrichtungen eingeladen an der Umfrage teilzunehmen. Die Institute/Behörden wurden über die jeweilige offizielle elektronische Anschrift per E‑Mail mit der Bitte zur Teilnahme an der Umfrage kontaktiert. Ein Informationsabschnitt zu Beginn der Umfrage klärte die Befragten über Ziel und Design der Studie und ihre Rechte auf.

Die Umfrage war über einen Zeitraum von 5 Monaten (Februar–Juni 2020) online verfügbar. Es wurden 2 Teilnahmeerinnerungen verschickt. Es wurde zudem eingeladen, Fragen und Wünsche zur Thematik, Feedback oder Kommentare über die Umfrage an die Organisierenden zu senden.

Für die folgende Auswertung wurden ausschließlich vollständig ausgefüllte/abgeschlossene Fragebögen berücksichtigt. Die Auswertung der Fragebögen erfolgte für die Themenfelder II_a_ und II_b_ anhand des angegebenen Erregerspektrums: In die Auswertung der erregerspezifischen Fragen zu phäno- und genotypischen Methoden wurden nur Fragebögen einbezogen, in welchen in der vorgeschalteten Filterfrage die Bearbeitung der Erreger angegeben wurde. Die Themen I, III, IV und V wurden erregerübergreifend ausgewertet.

Die Auswertung zum Erregerkomplex *Salmonella* wird in dieser Veröffentlichung dargestellt, die inhaltliche Auswertung zum Komplex STEC/EHEC findet sich in dem Beitrag von Richter et al. in diesem Themenheft.

## Ergebnisse

### Teilnehmende Laboratorien und deren Anwendungs- und Aufgabenbereiche

**Figure 1:**
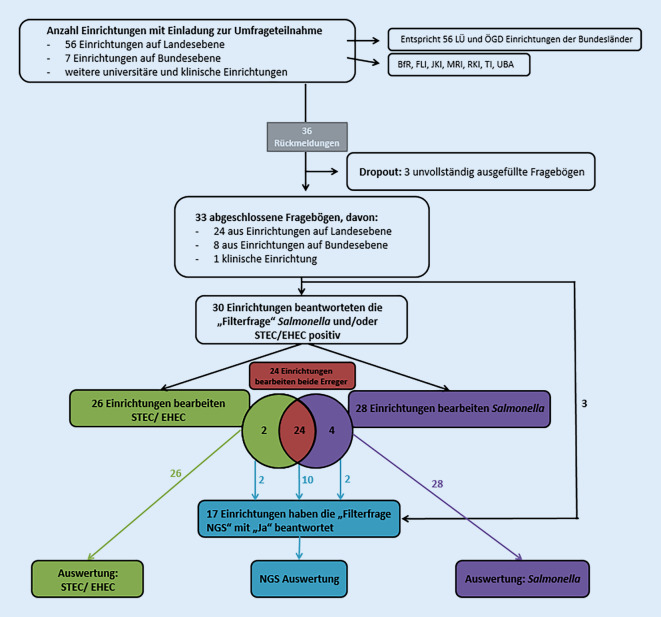

Die Einladung der Einrichtungen erfolgte über die Poststellen der Landesbehörden. Es wird davon ausgegangen, dass 40 LÜ- und 16 ÖGD-Einrichtungen auf Landesebene in Deutschland mit dem Anschreiben erreicht wurden. Zudem wurden auf Bundesebene das Robert Koch-Institut (RKI), Bundesinstitut für Risikobewertung (BfR), Friedrich-Loeffler-Institut (FLI), Julius Kühn-Institut (JKI), Thünen-Institut (TI), Max Rubner-Institut (MRI) und Umweltbundesamt (UBA) kontaktiert (Abb. 1). Von den insgesamt 63 eingeladenen Einrichtungen wurden 33 vollständig beantwortete Fragenbögen zur Auswertung herangezogen. Teilweise ausgefüllte Fragebögen und offensichtliche Dubletten (*n* = 3) wurden von der Auswertung ausgeschlossen. Da sich viele der 63 Einrichtungen in weitere Abteilungen aufteilen, konnte keine verlässliche Rückmeldequote ermittelt werden.

**Figure 2:**
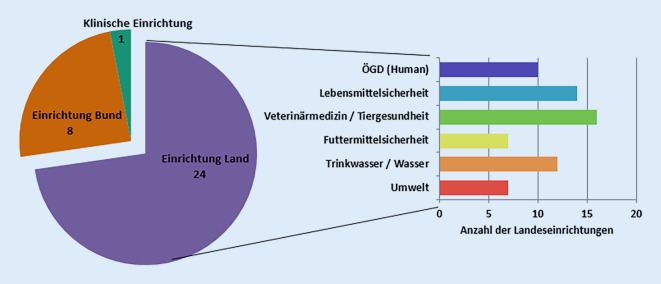

Hintergrundinformationen über die teilnehmenden Laboratorien wurden in der ersten Fragekategorie „Allgemeine Labor‑/Institutsinformation“ erhoben und analysiert. Die 33 Laboratorien ließen sich in unterschiedliche Sektoren auf Landesebene (*n* = 24) und Bundesebene (*n* = 8) sowie eine klinische Einrichtung unterteilen. Die Sektoren sind hierbei eingeteilt in ÖGD, LÜ, Veterinärmedizin/Tiergesundheit, Futtermittelsicherheit, Trinkwasser/Wasser und Umwelt. Sektorübergreifend beteiligten sich Einrichtungen aus 14/16 Bundesländern aus dem Bereich der staatlichen Untersuchungsämter im Veterinärwesen, der LÜ und des ÖGD an der Umfrage. Es war sowohl für den Sektor ÖGD als auch für den Sektor der LÜ eine Beteiligung von je 10 Bundesländern mit 10 bzw. 14 Einrichtungen (Abb. 2) zu verzeichnen.

Die 8 teilnehmenden Einrichtungen/Abteilungen auf Bundesebene deckten ebenso die humane, Lebensmitteluntersuchungs- und Tiergesundheitsüberwachungsseite ab. Eine Referenzfunktion auszuüben gaben 8 Einrichtungen an, davon 6 auf Bundesebene. Drei Einrichtungen waren als Nationales Referenzzentrum (NRZ) und 2 als Konsiliarlabor (KL) berufen, eine teilnehmende Einrichtung fungierte sowohl als NRZ und als KL. 2 weitere Einrichtungen waren Referenzlabor nach Verordnung (EG) Nr. 882/2004 bzw. 2017/625 sowie 2 andere Referenzlabor nach Tiergesundheitsgesetz. Über eine Akkreditierung verfügten 28/33 (27 nach DIN EN ISO 17025, 8 nach DIN EN ISO 15189, davon 7 nach DIN EN ISO 17025 und ISO 15189).

Häufigster Anlass für die Generierung von Typisierungsdaten waren epidemiologische Fragestellungen, die Aufklärung von Ausbrüchen, Überwachungs- und Monitoringprogramme, amtliche Kontrollen oder klinische Diagnostik. Weitere Verwendungszwecke der Analysen waren Forschungsprojekte, Betriebshygiene/Eigenkontrollen, Erreger-Surveillance und veterinärmedizinische Diagnostik.

Die Differenzierung der Teilnehmenden anhand des Erregerspektrums ergab, dass sich 28/33 mit *Salmonella*-Diagnostik befassen sowie 26/33 STEC/EHEC-verdächtige Proben bearbeiten und analysieren (Abb. 1).

### Bestandsaufnahme der verfügbaren und aktuell eingesetzten Typisierungsmethoden von *Salmonella enterica*

Von den teilnehmenden Einrichtungen befassten sich 28 mit der Diagnostik von *Salmonella enterica* und wurden daher in die „*Salmonella*-spezifische Auswertung“ einbezogen. Vier der teilnehmenden Institutionen waren Bundesbehörden, während es sich bei den restlichen 24 um Landesinstitutionen handelte. Die Zuordnung zu den verschiedenen Sektoren zeigte, dass 10/24 teilnehmende Einrichtungen im ÖGD, 14/24 in der LÜ und 16/24 im Bereich der Tiergesundheit aktiv sind. Da es Schnittmengen zwischen den Sektoren oder eine „One-Health“-Ausrichtung von Laboratorien/Einrichtungen gibt, war eine Mehrfachnennung von Sektoren für die Teilnehmenden möglich. Auf Bundesebene waren alle Sektoren abgedeckt. Die räumliche Zuordnung zeigte, dass sich aus dem ÖGD Laboratorien aus 10 Bundesländern, im Bereich LÜ Einrichtungen aus 9 Bundesländern und in der Veterinärmedizin/Tiergesundheit Institutionen aus 10 Bundesländern beteiligten. Die Teilnehmenden auf Bundesebene umfassten zudem das NRZ *Salmonella *am RKI, das Nationale Referenzlabor (NRL) *Salmonella* am BfR und das NRL Salmonellose der Rinder am FLI.

*Salmonella* stellte bei den teilnehmenden Institutionen in der Regel den am häufigsten bearbeiteten Erreger dar. In Summe werden von den teilnehmenden Instituten auf Landesebene pro Jahr mindestens 65.000 Proben mit *Salmonella*-Verdacht bearbeitet. Phänotypisch detaillierter untersucht werden hiervon mindestens 5700 Isolate, molekularbiologisch mindestens 100 Isolate. Auf Bundesebene kann von ca. 10.000 bearbeiteten *Salmonella*-Isolaten pro Jahr ausgegangen werden. Molekularbiologisch werden zudem ca. 4700 Isolate analysiert. Da nicht alle Teilnehmenden Angaben zur Anzahl der bearbeiteten Proben/Isolate machten, können diese Zahlen nur einen groben Überblick geben. Aufgrund der unterschiedlichen Spezialisierungen und Untersuchungstiefe der Laboratorien kann es zu Doppelungen bei den genannten Einsendungen von Isolaten auf Länder- und Bundesebene kommen.

Für den ÖGD gaben Laboratorien aus 3 Bundesländern 7100 bearbeitete humane *Salmonella*-Proben pro Jahr an, 870 Proben wurden aus 4 Bundesländern phänotypisch bearbeitetet. Für die verbleibenden Bundesländer konnte keine Differenzierung zwischen den verschiedenen Sektoren vorgenommen werden.

### Verfügbarkeit von Phäno- und Genotypisierungsverfahren zur Analyse von *Salmonella enterica*

Phänotypisch werden über die Bundes- und Landesinstitutionen mehr als 15.500 *Salmonella*-Isolate pro Jahr bearbeitet. Alle teilnehmenden Institutionen gaben an, phänotypische Verfahren zur Erregerdifferenzierung anzuwenden.

**Figure 3:**
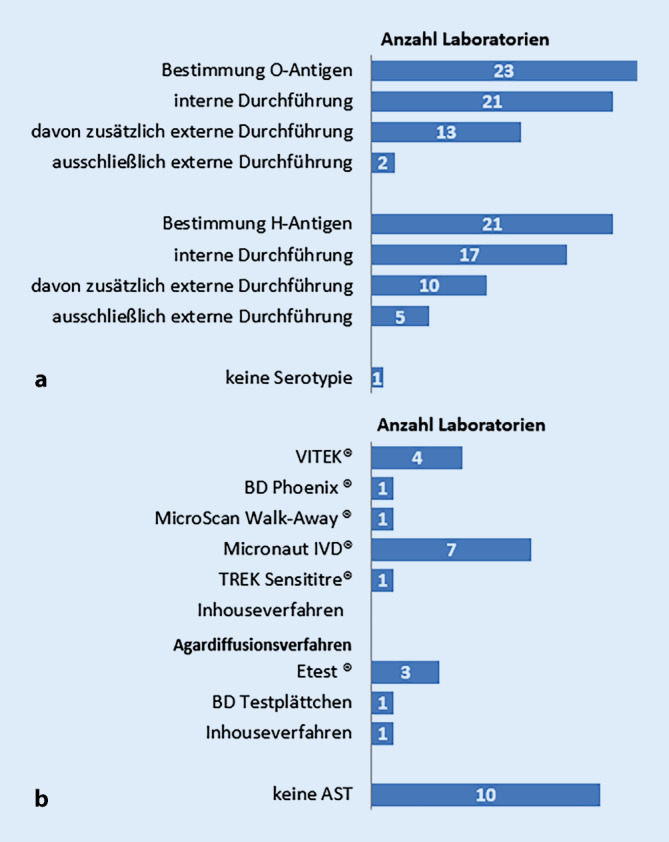

Häufigstes Verfahren war die Serotypie. Bis auf eine Einrichtung wird in allen teilnehmenden Landesinstitutionen die Serotypie von *Salmonella*-Erregern intern durchgeführt (*n* = 21) oder zusätzlich (*n* = 13) bzw. ausschließlich extern in Auftrag gegeben (*n* = 2; Abb. 3a). Einige Einrichtungen (*n* = 3) bestimmen intern ausschließlich die O‑Antigene, die H‑Phasen werden extern ermittelt. Einige Laboratorien, welche intern eine O- und H‑Antigenbestimmung durchführen, gaben ebenso an, zusätzliche Serotypie extern durchführen zu lassen. Es kann vermutet werden, dass die Vor-Ort-Bestimmung durch die Verfügbarkeit der Seren limitiert ist.

Auf Bundesebene führen die 3 in Deutschland etablierten Referenzlabore für Salmonellen die Serotypie vollumfänglich durch.

Weitere angegebene, häufig angewandte phänotypische Verfahren zur Erregeridentifizierung sind: „Bunte Reihe, API Teststreifen, Kligler und Vitek“. Zudem gaben 15/24 Landesinstitutionen an, MALDI-TOF-Massenspektrometrie (MS) zur Erregeridentifizierung einzusetzen.

Insgesamt 14/24 Laboratorien auf Landesebene führen antimikrobielle Empfindlichkeitstests durch. Zu diesem Zweck wird von allen Laboratorien das Verfahren der Mikrodilutionstests eingesetzt. Das häufigste Testsystem ist das Micronaut-System (7/14). Weitere angewandte Verfahren sind: VITEK, BD Phoenix, MicroScan Walk-Away und *Inhouse*verfahren (Abb. 3b). Die Interpretation der Ergebnisse der antimikrobiellen Empfindlichkeitstestungen erfolgt in 5 Fällen ausschließlich nach dem European Committee on Antimicrobial Susceptibility Testing (EUCAST) und in 3 Fällen nach dem Clinical and Laboratory Standards Institute (CLSI), in 2 Fällen nach beiden Leitlinien. 4 Einrichtungen machten keine Angaben.

Insgesamt gaben 7/24 Einrichtungen der Länder und 4/4 des Bundes an, molekularbiologische Typisierverfahren für die *Salmonella*-Diagnostik einzusetzen. Molekularbiologische Verfahren werden auf Landesebene hauptsächlich zur Speziesidentifizierung (PCR) genutzt. Die molekularbiologische Serovaridentifizierung wird dagegen mehrheitlich auf Bundesebene (NRZ/NRL) eingesetzt. Weitere molekularbiologische Verfahren spielen auf Landesebene keine größere Rolle, wogegen auf Bundesebene noch weitere Typisierverfahren vorgehalten werden (z. B. d‑Tartrat PCR, MVLA, PFGE, Plasmidtypisierung).

Die erwähnten phänotypischen und molekularbiologischen Methoden wurden von 8 Einrichtungen aus Bund und Ländern zur Ausbruchsuntersuchung oder Unterstützung dieser eingesetzt. In der Regel erfolgte die laborgestützte Ausbruchsaufklärung durch WGS einschließlich bioinformatischer Verfahren (Core Genome Multilocus Sequence Typing (cgMLST), Single-nucleotide-polymorphism(SNP)-basierte Mappingverfahren) und wird durch die oben angegebenen zusätzlichen molekularbiologischen Methoden unterstützt. Weitere Angaben zu Verfahren, welche bei der Ausbruchsaufklärung eine Rolle spielen, sind: Serotypisierung, PCR, Fourier-Transform-Infrarotspektrometer (FT-IR), PFGE, MALDI-TOF-MS-Subtypisierung.

Angaben zu Faktoren, die bei der phänotypischen und genotypischen Charakterisierung von *Salmonella* hinderlich sind bzw. eine effektive Typisierung verzögern, wurden qualitativ erhoben. Antworten hierzu beinhalteten unter anderem die Finanzierung („Kosten für genotypische Typisierungen bzw. den Aufbau dieser Methoden“) und Schwierigkeiten bei der Serotypie („Analyse von Rauformen, monophasischen Varianten, seltenen Serovare (da nicht alle Antiseren vorrätig sind), nicht darstellbare 2. H-Phase“).

### Zugang zur WGS, Bioinformatik und Verwaltung der Typisierdaten

Die Auswertung zur Verfügbarkeit der WGS, Bioinformatik und Datenverwaltung erfolgte für alle Konsortien erregerübergreifend. Die Teilnehmenden wurden befragt, in welchem Rahmen ein Zugang zu und die Anwendung von WGS-Verfahren sowie Kapazitäten und Expertise für anschließende bioinformatische Auswertungen intern bzw. extern vorhanden sind.

Insgesamt gaben 17/33 Einrichtungen an, über interne (*n* = 14) oder externe (*n* = 3) Kapazitäten Zugang zur WGS zu haben, 5 Einrichtungen haben keine Angabe gemacht. Von den 17 Laboratorien mit WGS-Zugang sind 8 den staatlichen Laboratorien des Bundes zuzuordnen, 8 weitere den Laboratorien der Länder und einer klinischen Einrichtung. Anzumerken ist, dass unter Laboratorien mit WGS-Zugang 7 mit Referenzfunktion wiederzufinden waren.

**Figure 4:**
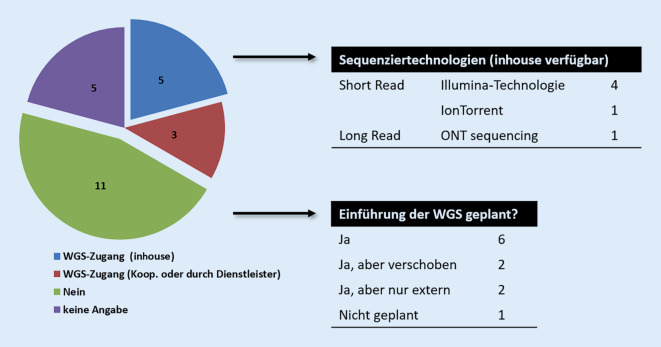

Während alle Einrichtungen auf Bundesebene Zugang zu WGS haben, gaben von den Teilnehmenden auf Landesebene nur 8/24 WGS-Kapazitäten an (33 %, Abb. 4). Zehn der Laboratorien auf Landesebene planten eine interne Etablierung oder externe Nutzung von WGS in absehbarer Zeit (innerhalb der nächsten 3 Jahre). Akute Hindernisse, welche einer Einführung von WGS-Technologien entgegenstehen, wurden mit Personal- und Finanzierungsengpässen angegeben.

In 16 der 17 Einrichtungen mit Zugang zur WGS wurde die Illumina-Plattform zur Sequenzdatengenerierung genutzt, in 4 Laboratorien stand ein Ion-Torrent-Gerät zur Verfügung. Die Anwendung von* Long-read-sequencing*-Technologien wurde hauptsächlich durch die Oxford Nanopore Technologie (ONT, *n* = 8) abgedeckt. Nur ein teilnehmendes Laboratorium berichtete über den Zugang zum SMRT Sequencing („PacBio“-Sequenziergerät).

Die Nutzung der WGS-Daten erfolgte vorrangig zur Erfüllung von Surveillance-Aufgaben und Ausbruchsuntersuchungen. Hierbei wurde vom Großteil der Einrichtungen eine routinemäßige Sequenzierung von ausgewählten Isolaten angestrebt, um epidemiologische Zusammenhänge bei Ausbruchsgeschehen, unklare Routineproben oder Stämme mit besonderen Phänotypen zu untersuchen. Eine vollständige Sequenzierung aller Isolate wurde bei einigen Einrichtungen angestrebt, aber bisher nicht erreicht. Weiterhin standen insbesondere die Antibiotikaresistenz- und Virulenzgen-Vorhersage, *In-silico-*Serotypie sowie die Typisierung von Erregern mittels cgMLST oder SNP-Analysen im Vordergrund. Bei der Sequenzdatenauswertung kamen neben öffentlich zugänglichen Tools (CGE Finder Series, ABRicate, SeqSero) auch kommerzielle Plattformen zur Anwendung (Ridom SeqSphere^+^, 9/17 Laboratorien). Der Einsatz von WGS zu weitergehenden Forschungszwecken erfolgte hauptsächlich in Einrichtungen auf Bundesebene.

Die Verwaltung von WGS-Rohdaten wurde bei fast allen teilnehmenden Laboratorien auf eigenen Datenbanken/Servern realisiert. Eine Freigabe der Daten erfolgte (teilweise) über öffentlich zugängliche Repositorien (ENA, NCBI, SRA, Enterobase, nach Publikationen). Die Vorgaben zur Speicherdauer von WGS-Rohdaten waren bei den Einrichtungen unterschiedlich, i. d. R. werden die Daten aber für mindestens 5 Jahre aufbewahrt.

Die Verwaltung von WGS-Analyseergebnisdaten wurde bei den meisten Laboratorien entweder über kommerzielle Plattformen (Ridom SeqSphere^+^), *Inhouse*datenbanksysteme/LIMS oder Tabellenkalkulationsprogramme (Excel) realisiert.

Der wichtige Aspekt zur Verfügbarkeit/Freigabe von Analyseergebnisdaten und Rohdaten für die (Fach‑)Öffentlichkeit wird nach Ansicht vieler Einrichtungen kritisch gesehen, da häufig Unsicherheiten über die Weitergabe und Besitzrechte generierter Sequenzdaten und die rechtliche Grundlage zur Datenfreigabe bestehen. Zudem werden Daten durchaus bis zur Veröffentlichung von Berichten und Publikationen zurückgehalten. Behördenübergreifende Anfragen bei der Zusammenarbeit zur Ausbruchsaufklärung sind von diesen Beschränkungen jedoch ausgenommen.

### Schulungsbedarf

Ein wichtiger Aspekt zur Etablierung von WGS-Technologien ist der Zugang zur bioinformatischen Expertise. Da im Rahmen der BMG-Projekte im Themenfeld „Integrierte genombasierte Surveillance von zoonotischen Erregern und Erregern mit speziellen Antibiotikaresistenzen“ die Durchführung von Workshops und die Erstellung von Schulungsmaterial erfolgen wird, wurde der Bedarf der Teilnehmenden nach Schulungen mit bioinformatischem Schwerpunkt abgefragt. Da die WGS bei den teilnehmenden Laboratorien auf Bundesebene schon etabliert ist, wird hier auf das Feedback der Landeslaboratorien eingegangen.

Auf die Frage, in welchem Bereich noch Schulungsbedarf besteht, haben die Einrichtungen insbesondere grundlegende und erweiternde Fortbildungen zu bioinformatischen Analysen angegeben (11/24). Ein weiterer Punkt ist die Integration/Interpretation von WGS-Daten zur Ausbruchsuntersuchung (10/24). Qualitative Antworten umfassten zudem die Validierung von Verfahren und Ergebnissen sowie Limitationen der WGS-Analysen im Hinblick auf ihre Aussagekraft.

## Diskussion und Schlussfolgerungen

Erkenntnisse aus der sektorenübergreifenden molekularen Surveillance von zoonotischen Erregern stellen eine wichtige Grundlage zur Identifizierung und Aufklärung von (lebensmittelbedingten) Ausbruchsgeschehen und der Verbreitung von antimikrobiellen Resistenzdeterminanten dar. Die Informationen dieser Verfahren können in Kombination mit epidemiologischen Daten dazu beitragen, Quellen von Ausbruchsgeschehen schneller und verlässlicher zu identifizieren und somit Kontrollmaßnahmen zielgerichteter einzuleiten. Insbesondere in den vergangenen 5–6 Jahren hat sich der Stand der Technik so entwickelt, dass genombasierte Typisierungsverfahren bisherige molekulare Methoden (z. B. PFGE und MVLA) bei Überwachungs- und Ausbruchsuntersuchungen ersetzen. Im internationalen Kontext stellen WGS-basierte Typisierverfahren zunehmend den „Goldstandard“ dar [1, 7].

Umfragen unter den nationalen Referenzlaboratorien des öffentlichen Gesundheitswesens der Europäischen Mitgliedsstaaten in den Jahren 2015 und 2016 legten den Fokus auf Zugang zur WGS und die operative WGS-basierte Typisierungskapazität für die nationale Überwachung ausgewählter lebensmittelbedingter Krankheitserreger. Die Ergebnisse konnten zeigen, dass der Großteil der Referenzlaboratorien Zugang zur WGS-basierten Typisierung hatte und der weitere Ausbau der Kapazitäten vorangetrieben wurde [6].

Unsicherheit herrschte dagegen bisher, in welchem Rahmen auf nationaler Ebene WGS-basierte Verfahren von den verschiedenen Stakeholdern angewandt werden. Aufgrund des zoonotischen Charakters lebensmittelbedingter Ausbrüche und der Verbreitung von antimikrobiellen Resistenzdeterminanten ist es für die Bestrebungen einer WGS-gestützten Surveillance für Infektionserreger zudem von großer Bedeutung, einen sektorübergreifenden *One-Health*-Ansatz zu verfolgen. Aktuelle Bemühungen in den verschiedenen Sektoren haben zum Ziel, molekulare Surveillance-Technologien soweit zu synchronisieren, dass ein detaillierter Datenaustausch und Kommunikation in Zukunft stattfindet [8, 9]. Die hier ausgewertete Befragung sollte daher einen Einblick geben, inwiefern Typisierungsmethoden für die zoonotischen Erreger *Salmonella,* STEC/EHEC und Erreger mit speziellen Antibiotikaresistenzen sowie WGS-Technologien und Auswertekapazitäten auf nationaler bzw. Länderebene angewendet werden oder in Zukunft verfügbar sind. Außerdem sollte identifiziert werden, wo noch spezieller Nachhol- und Unterstützungsbedarf besteht.

Positiv anzumerken war die hohe Beteiligung der staatlichen Laboratorien der Länder und des Bundes, welche mit der Typisierung von lebensmittelassoziierten Erregern eine offizielle Funktion bei der Aufklärung und Verfolgung von Ausbruchsgeschehen ausüben. Anhand der Umfrageergebnisse konnte gezeigt werden, dass bisher einige Landeslaboratorien die WGS in ihre täglichen Routinen implementierten oder extern anwandten (*n* = 8).

Interessant war, dass viele weitere teilnehmende Institute angaben, in Zukunft zu planen, intern oder aber auch extern WGS-Technologien einzusetzen (10/11 der Teilnehmenden mit Angabe, WGS sind bisher nicht implementiert). Wie bereits aus anderen Umfragen bekannt war, zeigte sich auch hier, dass Laboratorien auf Bundesebene die WGS schon häufig in die täglichen Routinen implementiert hatten [10, 11].

Zu beachten ist, dass die Ergebnisse der Umfrage den Stand der Laboratorien zur Implementierung und Ausbau der molekularen Surveillance zum Zeitpunkt „Prä-Corona“ darstellen (Umfragezeitraum: Februar–Juni 2020). Im Zuge der COVID-19-Pandemie kam es zu einer Intensivierung der Sequenzierinitiativen. Zur Sequenzierung befähigte Einrichtungen könnten daher ihre bereits etablierten Kapazitäten mit Unterstützung ausbauen oder hochskalieren und neben der COVID-19-Diagnostik (oder nach der Pandemie) die Sequenzierung von zoonotischen Erregern realisieren.

Am Beispiel der NRZ und NRL für *Salmonella* lässt sich gut die Intensivierung der Sequenzierkapazitäten nach Implementierung und Etablierung betrachten. In beiden Laboratorien fand die Technologieeinführung im Jahr 2014/2015 statt. Im Anschluss folgten Validierungsphase und der Einsatz der Technologie in der Routine und ein steter Anstieg der Sequenzier- und Analysekapazitäten. Zusätzlich wurden schrittweise immer weitere Erreger in das Sequenzier- und Analysespektrum mit aufgenommen.

Neben dem „reinen“ Ausbau der Sequenzierkapazitäten stellt die Befähigung zur Datenauswertung einen essenziellen und kritischen Punkt dar. Geschultes Personal und einheitliche Standards in Methodik und Dateninterpretation sind für eine sektorübergreifende Kommunikation unerlässlich. Die bereits sequenzierenden teilnehmenden Laboratorien gaben an, auf *Inhouse*lösungen, öffentliche Module (z. B. Enterobase, CGE Series) sowie auch auf kommerzielle Projektpakete (z. B. Ridom SeqSphere^+^) zurückzugreifen. Diverse Analyseplattformen und damit verbundene Nomenklaturen (EnteroBase, Ridom SeqSphere^+^, SNP *addresses*) wie sie z. B. für die *Salmonella*-Diagnostik auf europäischer Ebene angewandt werden, erschweren einen schnellen Austausch und die Kommunikation zwischen den Partnern. Eine einheitliche Lösung für Deutschland sollte daher angestrebt werden.

## Fazit

Eine WGS-basierte Erregertypisierung wird als die Methode mit der höchsten Trennschärfe und bestmöglichen Standardisierbarkeit (Diskriminierungsfähigkeit) zur Erkennung von Infektionsclustern anerkannt. Ihre Verwendung zu diesem Zweck wird vom European Centre for Disease Prevention and Control (ECDC) und von der Europäischen Behörde für Lebensmittelsicherheit (EFSA) empfohlen und sollte in diesem Bereich den Goldstandard darstellen [1, 7]. Die hier vorgestellte Umfrage zeigte, dass viele der teilnehmenden Laboratorien über einen hohen Anteil an molekularen Methoden zur Identifikation bzw. Typisierung von Zoonoseerregern verfügen und in einigen Fällen schon eine Implementierung der WGS stattgefunden hat. Die Illumina-Sequenzierung ist hierbei die am weitesten verbreitete Technologie. Des Weiteren gibt es zahlreiche Laboratorien, die eine Implementierung der WGS-Technologien einschließlich bioinformatischer Auswertemöglichkeiten anstreben. Für einen erfolgreichen Aufbau und den nutzungsorientierten und sektorübergreifenden Einsatz der WGS sind daher ein gemeinsamer Wissens- und Erfahrungsaustausch und der Einsatz von identischer Software oder Software mit kompatiblen Schnittstellen empfehlenswert. Schulungen und Schulungsmaterialien, welche im Rahmen der 3 Konsortien zur Verfügung gestellt und angeboten werden, können dabei unterstützen.

### Infobox 1 Konsortien im Themenfeld „Integrierte genombasierte Surveillance von zoonotischen Erregern und Erregern mit speziellen Antibiotikaresistenzen“

**GenoSalmSurv (Integrierte genombasierte Surveillance von Salmonellen)**

Im Projekt GenoSalmSurv liegt der Fokus auf dem Aufbau einer integrierten genombasierten Surveillance des zoonotischen Erregers *Salmonella *spp. Zu diesem Zweck werden harmonisierte Verfahren unter Mitwirkung der Institutionen des Öffentlichen Gesundheitsdienstes (ÖGD) des Bundes und der Länder sowie der Einrichtungen für Lebensmittelsicherheit und Tiergesundheit generiert. Die wichtigsten Ziele des Projekts sind daher die Identifizierung und Erstellung von Standardverfahren für die gemeinsame Nutzung und Analyse von Ganzgenom-Daten von *Salmonella-*Isolaten der verschiedenen Sektoren sowie die Bereitstellung von bioinformatischen Open-Source-Tools für die anschließende Datenanalyse, welche Stammvergleiche von Salmonellen mit einer hohen Auflösung ermöglicht. Die Ergebnisse und Arbeitsmaterialien werden zur weiteren Nutzung dem öffentlichen Gesundheitsbereich bereitgestellt und in entsprechenden Schulungen und Veranstaltungen vermittelt (Projektwebsite: https://www.rki.de/DE/Content/Institut/OrgEinheiten/Abt1/FG11/Projekt_GenoSalmSurv.html).

**IGS-Zoo (Integrierte Genomische Surveillance von Zoonoseerregern)**

Im Rahmen des Projekts IGS-Zoo erfolgt die Entwicklung von Konzepten für eine sektorübergreifende genomische Surveillance von Shiga-Toxin-bildenden bzw. enterohämorrhagischen *Escherichia* (*E*.)* coli* (STEC/EHEC) in Deutschland. Dadurch können eine Verbesserung der Patientenversorgung und eine Erhöhung der Lebensmittelsicherheit im Sinne von *One Health* erreicht werden. Neben der rein technischen Umsetzung der Genomsequenzierung und der nachfolgenden bioinformatischen Analysen ist für eine erfolgreiche und effektive Nutzung einer genomischen Surveillance ein hohes Maß an Kommunikation und Abstimmung zwischen den verschiedenen Sektoren entscheidend. Hierzu sollen deshalb Standards für die Nutzung einer integrierten genomischen Surveillance durch den ÖGD und die Lebensmittelüberwachung (LÜ) sowie Standards zur Datenharmonisierung entwickelt werden. Schließlich sollen Schulungsmaterialien zur Unterstützung der Einführung einer integrierten genomischen Surveillance im ÖGD und in der LÜ erstellt werden (Projektwebseite: http://www.igs-zoo.org).

**GÜCCI (Genombasierte Surveillance übertragbarer Colistin- und Carbapenemresistenzen Gram-negativer Infektionserreger)**

Infektionen mit Carbapenem- und/oder Colistin-resistenten Enterobacterales (*E. coli, Klebsiella pneumoniae* u. a.) sind gefürchtete Komplikationen, vor allem bei stationären, schwer kranken und älteren Patientinnen und Patienten. Eine Carbapenemresistenz wird häufig durch Enzyme, die Carbapenemasen vermittelt. Colistin ist eine wichtige Therapieoption bei Infektionen mit carbapenemaseproduzierenden Enterobacterales. Colistin kann im Verlauf einer Therapie seine Wirksamkeit durch die Selektion resistenter Bakterien, aber auch durch einen Erwerb von Resistenzgenen vom *mcr*-Typ verlieren. Sowohl die Gene für Carbapenemasen als auch Colistinresistenz sind häufig mobil und verbreiten sich somit sowohl klonal als auch horizontal. Mit dem Projekt GÜCCI wird modellhaft die dringend notwendige Implementierung einer Kerntechnologie für eine genombasierte Erreger- und Resistenz-Surveillance in Leitinstitutionen des ÖGD und Veterinärwesens und der epidemiologisch adäquaten Aus- und Bewertung realisiert (Projektwebseite: https://www.rki.de/DE/Content/Institut/OrgEinheiten/Abt1/FG13/guecci.html).

## References

[1] European Centre for Disease Prevention and Control (2016). Expert opinion on whole genome sequencing for public health surveillance.

[2] Besser J, Carleton HA, Gerner-Smidt P, Lindsey RL, Trees E (2018). Next-generation sequencing technologies and their application to the study and control of bacterial infections. Clin Microbiol Infect.

[3] Mellmann A, Andersen PS, Bletz S (2017). High interlaboratory reproducibility and accuracy of next-generation-sequencing-based bacterial genotyping in a ring trial. J Clin Microbiol.

[4] Schurch AC, van Schaik W (2017). Challenges and opportunities for whole-genome sequencing-based surveillance of antibiotic resistance. Ann N Y Acad Sci.

[5] Bundesinstitut für Risikobewertung (2020). Anwendung des Whole Genome Sequencing zur Aufklärung von lebensmittelbedingten Krankheitsausbrüchen. BfR-Wissenschaft.

[6] Revez J, Espinosa L, Albiger B (2017). Survey on the use of whole-genome sequencing for infectious diseases surveillance: rapid expansion of European national capacities, 2015–2016. Front Public Health.

[7] Koutsoumanis K, Allende A, EFSA Panel on Biological Hazards (EFSA BIOHAZ Panel) (2019). Whole genome sequencing and metagenomics for outbreak investigation, source attribution and risk assessment of food-borne microorganisms. EFS2.

[8] Uelze L, Becker N, Borowiak M (2021). Toward an integrated genome-based surveillance of salmonella enterica in Germany. Front Microbiol.

[9] MyGenomeSurv (2021) www.miGenomeSurv.org. Zugegriffen: 01.07.2021

[10] Control ECfDPa (2018). Monitoring the use of whole-genome sequencing in infectious disease surveillance in Europe.

[11] Malorny B, Scheel K, Rau J (2020). Onlineumfrage zur Anwendung von molekularbiologischen Typisierungsverfahren und MALDI-TOF-MS in diagnostischen Laboren in Deutschland. J Consumer Prot Food Saf.

